# Attribute analysis and modeling of color harmony based on multi-color feature extraction in real-life scenes

**DOI:** 10.3389/fpsyg.2022.945951

**Published:** 2022-09-14

**Authors:** Shuang Wang, Jingyu Liu, Jian Jiang, Yujian Jiang, Jing Lan

**Affiliations:** ^1^State Key Laboratory of Media Convergence and Communication, Communication University of China, Beijing, China; ^2^Key Laboratory of Acoustic Visual Technology and Intelligent Control System, Ministry of Culture and Tourism, Communication University of China, Beijing, China; ^3^Beijing Key Laboratory of Modern Entertainment Technology, Communication University of China, Beijing, China; ^4^China Digital Culture Group Co., Ltd., Beijing, China; ^5^Center for Ethnic and Folk Literature and Art Development, Ministry of Culture and Tourism, Beijing, China

**Keywords:** color harmony, aesthetic measure, multiple colors, feature extraction, machine learning, real-life scene

## Abstract

Color harmony is the focus of many researchers in the field of art and design, and its research results have been widely used in artistic creation and design activities. With the development of signal processing and artificial intelligence technology, new ideas and methods are provided for color harmony theory and color harmony calculation. In this article, psychological experimental methods and information technology are combined to design and quantify the 16-dimensional physical features of multiple colors, including multi-color statistical features and multi-color contrast features. Eighty-four subjects are invited to give a 5-level score on the degree of color harmony for 164 multi-color materials selected from the screenshots of film and television scenes. Based on the multi-color physical features and the subjective evaluation experiment, the correlation analysis is firstly carried out, which shows that the overall lightness, difference of the color tones, number of multiple colors, lightness contrast, color tone contrast, and cool/warm contrast are significantly correlated with color harmony. On the other hand, the regression prediction model and classification prediction model of color harmony are constructed based on machine learning algorithms. In terms of regression prediction model, the prediction accuracy of linear models is higher than that of nonlinear models, with 63.9% as the highest, indicating that the multi-color physical features can explain color harmony well. In terms of classification prediction model, the Random Forest (RF) has the best prediction performance, with an accuracy of 80.2%.

## Introduction

Color harmony refers to the color matching of two or more colors, organized in an orderly and coordinated manner, which can make people feel happy and satisfied (Zheng, 2013). Color harmony is widely used in industrial design, graphic design, interior design, and other color-matching scenes, and its strong correlation with emotion is further applied in computer vision fields such as affective computing and semantic analysis.

Color harmony theory is divided into classic color harmony theory and modern color harmony theory according to different analysis methods. In the 19th century, the study of classical color harmony theory focused on the qualitative analysis that was, color harmony theory was summed up in some descriptive color harmony rules, which were represented by Zheng (2013). Goethe believes that color can result in emotional fluctuations and expound the effect of color from physiological and psychological perspectives (Die Berlin-Brandenburgische Akademie der Vissenschaften in Göttingen und die Heidelberger Akademie der Wissenschaften (Hrsg.), 2004). Chevreul divides the color harmony rules into two categories, namely similar harmony and contrast harmony, which inspires the development of the color system research (Zhang et al., 1996). On this basis, Itten theorizes seven types of color contrast, including contrast by hue, contrast by value, contrast by temperature, contrast by complements (neutralization), simultaneous contrast (from Chevreuil), contrast by saturation (mixtures with gray), and contrast by extension (from Goethe) (Itten, 1999). However, these studies rely on fragmentary understanding of subjective experience and lack of data support, and most of them only stay on the properties of the research objects.

At the beginning of the 20th century, with the construction of the color system, the study of color harmony theory transformed into the quantitative analysis. Researchers tried to investigate, analyze, and explain color harmony, not only satisfied with the general rules of color harmony, but also committed to use a more precise way to elaborate the content of color harmony, which were represented by Munsell, Ostwald, Moon, and Spencer. Munsell develops the Munsell color system, which is the most famous color system so far, and then he proposes seven rules about color harmony, namely vertical harmony, radial harmony, circumferential harmony, oblique interior harmony, oblique transverse interior harmony, spiral harmony, and elliptical harmony (Zhang and Zhang, 2003). The color harmony theory of Ostwald is composed by the regular positions in his color system, including two-color harmony, three-color harmony, and multi-color harmony (Sakahara, 2002). Based on the Munsell color system, Moon and Spencer make a comprehensive qualitative study on color harmony, including the geometric formulation of classical color harmony theory, area of color harmony, and evaluation on the degree of color harmony (Moon and Spencer, 1944).

Nowadays, with the development of signal processing and artificial intelligence, more and more researchers try to adopt different perspectives and methods for color harmony study.Lu et al. (2015) combine classical color harmony theory with machine learning algorithms, and propose a Bayesian framework for constructing color harmony model. In this framework, Matsuda color harmony model and Moon-Spencer color harmony model are integrated into the training process based on the Latent Dirichlet Allocation (LDA) as the prior condition, which achieves a good predicting performance on public data set. On this basis, Lu et al. (2016) further considers the spatial position relationship between each color and proposed a discriminant learning method based on LDA. Zaeimi and Ghoddosian (2020) proposed an art-inspired meta-heuristic algorithm to solve the global optimization for color harmony modeling. According to the relative position in the hue ring and the color harmony template in Moon-Spencer color harmony theory, this algorithm searched for and matched the best combination mode of multiple colors. Wang et al. (2022) combined experimental psychology with artificial intelligence technology together, divided the factors affecting color harmony into objective factors and subjective factors, studied the relationship among three of them, and constructed the mathematical model to predict color harmony. Her study firstly abstracted the generation process of color harmony from objective factors to subjective factors, and tried to construct a research paradigm of color harmony.

In general, the current research on color harmony modeling involves key technologies, such as feature extraction and machine learning. In terms of feature extraction Huang et al. (2022), proposed a Feature Map Distillation (FMD) framework under which the feature map size of teacher and student networks was different. Zhang et al. (2021) integrated several well-proved modules together to learn both short-term and long-term features from video inputs and meanwhile avoid intensive computation. In terms of machine learning, Ohata et al. (2021) propose an automatic detection method for COVID-19 infection based on chest X-ray images, which combines CNNs with consolidated machine learning methods, such as k-Nearest Neighbor, Bayes, Random Forest, multi-layer perceptron (MLP), and Support Vector Machine (SVM). Lei et al. (2022) propose a novel Meta Ordinal Regression Forest (MORF) method for medical image classification with ordinal labels, which learns the ordinal relationship through the combination of convolutional neural network and differential forest in a meta-learning framework, in order to improve model generalization with ordinal information. All the methods mentioned above can provide technology support for the current color harmony modeling research.

The existing research on color harmony theory mainly take simple multi-color combination as the research object, such as color pair and three-color combination, which cannot meet the application requirements of real-life scenes. Therefore, this article takes the multi-color materials in real-life scenes as the research object, combines experimental psychology with information technology together, and constructs a few-shot color harmony modeling method which is suitable for real-life scenes. To sum up, the chapter content arrangement is below. (1) Construct the dataset of multi-color materials and smooth the materials to eliminate the interference of the semantic information (see section “Multi-color dataset construction”). (2) Design and quantify the multi-color physical features (see section “Multi-color physical features extraction”). (3) Carry out the subjective evaluation experiment on color harmony based on the semantic difference (SD) method (see section “The subjective evaluation experiment on color harmony”). (4) Conduct data analysis based on the experimental results, and construct the multi-color harmony prediction model based on machine learning algorithms (see section “Results and discussion”).

## Multi-color dataset construction

### Multi-color materials acquisition

The multi-color materials should conform to the multi-color relationships in real-life scenes as much as possible, and the color-matching mode is supposed to be as rich as possible. Therefore, this article selects the screenshots of the film and television scenes based on real life as the source of multi-color materials, which are more practical than the synthetic ones (Jiang et al., 2019). The selecting principles are as follows: (1) color pictures; (2) remove the pictures containing virtual scenes in order to conform to the multi-color relationships of real-life scenes as much as possible; (3) remove the pictures containing obvious semantic information to avoid the impact on the evaluation of the degree of color harmony, such as expressions and words; and (4) select the pictures containing rich color information as much as possible. Therefore, we finally construct the multi-color dataset containing a total of 164 materials captured from the film and television scenes, which are taken from 18 classic film types with a resolution of 1,280 dpi × 720 dpi.

According to CIE Publication No. 17.4, hue is an attribute of a visual sensation according to which an area appears to be similar to one, or to proportions of two, of the perceived colors. Chroma is the colorfulness of an area judged in proportion to the brightness of a reference white. Lightness is the brightness of an area judged relative to the brightness of a reference white. Therefore, CIELAB color space (L* represents lightness, a* represents red/green, and b* represents yellow/blue) proposed by the International Commission on Illumination (CIE), which is perceptual uniform and device independent, is adopted to extract the basic color attributes (Connolly and Fleiss, 1997). Among them, we select the L* dimension to represent lightness, and the *h*/C* dimension which calculated by the a* dimension and b* dimension to present hue angle/chroma.

It is noted that the L*, a*, and b* values of CIELAB color space are obtained from the digital files, which are converted from RGB color space. The steps are as below based on ITU-R Recommendation BT.709 using the D65 white point reference.

Step I: Convert RGB color space to XYZ color space with the *R*, *G*, and *B* values normalized to the interval [0,1] .


(1) $$\left[\begin{array}{c} X\\ Y\\ Z \end{array}\right]=\left[\begin{array}{ccc} 0.412453 & 0.357580 & 0.180423\\ 0.212671 & 0.715160 & 0.072169\\ 0.019334 & 0.119193 & 0.950227 \end{array}\right]\left[\begin{array}{c} R\\ G\\ B \end{array}\right]$$

Step II: Normalize for D65 white point.


(2) $$X=\frac{X}{0.950456}$$

(3) $$Y=\frac{Y}{1.0}$$

(4) $$Z=\frac{Z}{1.088754}$$

Step III: Calculate the value of f(t) , where t represents X , Y , or Z value of XYZ color space.


(5) $$f\left(t\right)=\{\begin{array}{c} t^{1/3},if\;t>{\left(\frac{6}{29}\right)}^{3}\\ \left(\frac{1}{3}\right){\left(\frac{29}{6}\right)}^{2}t+\frac{16}{116},others \end{array}$$

Step IV: Convert XYZ color space to CIELAB color space.


(6) $$L^{*}=116f\left(Y\right)-16$$

(7) $$a^{*}=500\left(f\left(X\right)-f\left(Y\right)\right)$$

(8) $$b^{*}=200\left(f\left(Y\right)-f\left(Z\right)\right)$$

**Figure 1:**
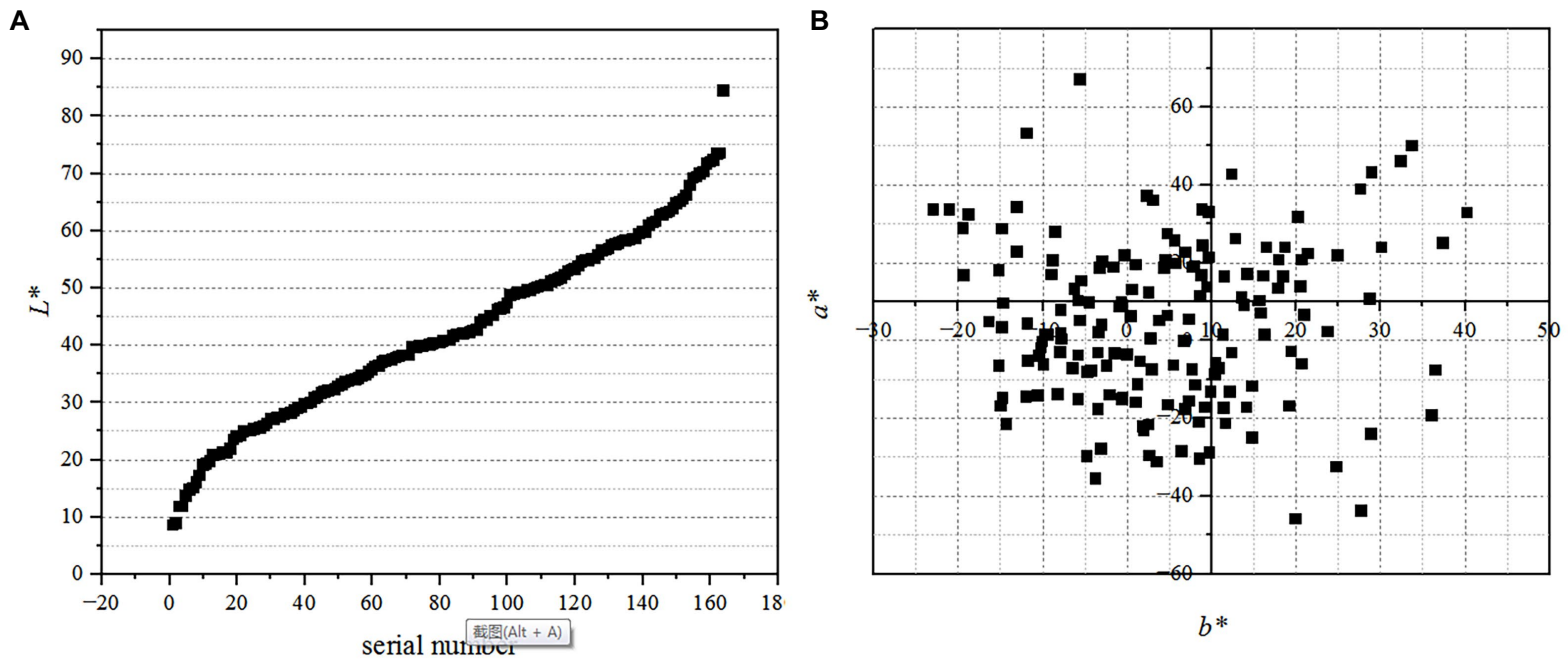
The spatial distribution of the color basic attributes of materials. Among them, **(A)** shows the distribution of L* value with *X*-axis representing the serial number of L* value ranging from smallest to largest, and **(B)** shows the plane distribution of a* and b* value.

Finally, the spatial distribution of the color basic attributes materials is shown in Figure 1.

### Multi-color materials pre-processing

The materials are taken from the screenshots of film and television scenes, which not only contain color information, but also contain edge information. In general, people recognize specific objects by edge information, which also contains a lot of semantic information, and has an impact on the evaluation on the degree of color harmony. Therefore, it is necessary to process the edge information of multi-color materials.

A circular average filter is adopted to smooth the edge information of multi-color materials. The main idea of the average filter is to replace the grayscale of a pixel with the average grayscale of other pixels in the neighborhood. The steps are below.

Step I: Set the grayscale of pixel (i,j) as g(i,j) .

Step II: Then, calculate the average value $\underset{\left(x,y\right)\in \Omega }{\sum }\bar{g\left(x,y\right)}$ of all pixels in the circular template Ω , with (i,j) as the center and *n* as the radius.

Step III: Finally, replace the grayscale g(i,j) of the originalpixel (i,j) with the average value $\underset{\left(x,y\right)\in \Omega }{\sum }\bar{g\left(x,y\right)}$ .

This article set the smoothing degree *n* as 50 pixels according to the subjective evaluation experiment carried out by the previous study (Wang et al., 2020). In this experiment, two multi-color materials with rich edge information are selected from each color tone category as evaluation materials, and, respectively, smoothed by 10 circular mean filters, with the radius of each circular template ranging from 10 pixels to 100 pixels (10 pixels apart). The subjects are asked to evaluate the minimum smoothing radius in which edge information could not be seen in each group of materials.

**Figure 2:**
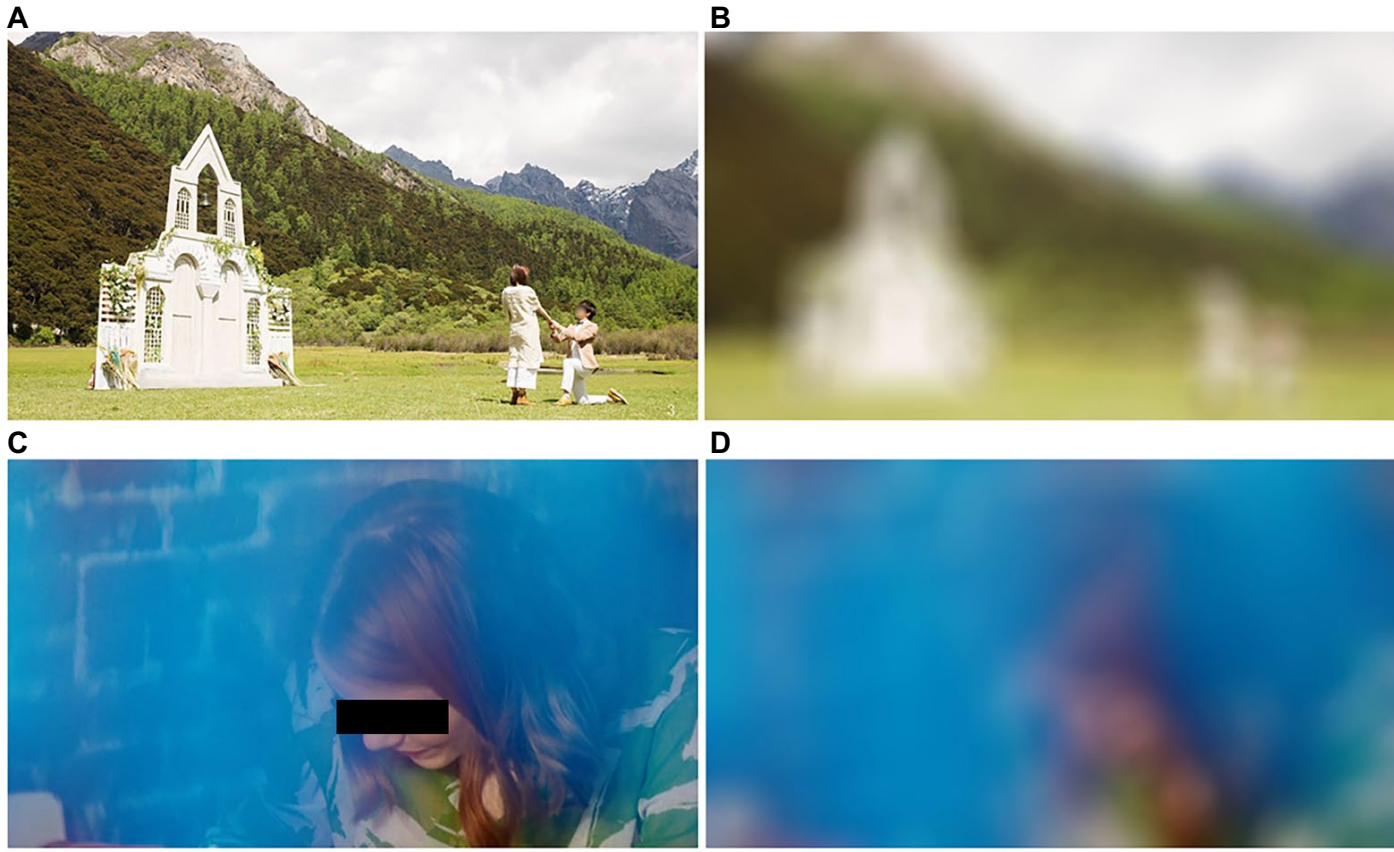
The differences between the original material and the smoothed material. Among them, **(A)** shows the original material, **(B)** shows the smoothed one, **(C)** shows the original material, and **(D)** shows the smoothed one.

Figure 2 shows the differences between the original material and the smoothed material. As shown in Figures 2A,B, the scene information in the original material (e.g., church) is blurred and cannot be effectively recognized by human eye. Similarly, as shown in Figures 2C,D, the facial information of the character is blurred, and you can never know whether the actor is the one you like or not.

### Color palette construction

In general, color palette refers to the board used to harmonize the fresh pigment, which can be divided into circular palette, elliptic palette, and rectangular palette according to the shape. In color analysis of film and television scenes, the rectangular color palette is often adopted to display the dominant colors and their proportion information. Therefore, in order to enable the subjects to master the multi-color information of the materials more intuitively, we first construct the rectangular color palette of multi-color materials based on K-means clustering algorithm (Krishna and Murty, 1999). $\left\{x^{\left(1\right)},x^{\left(2\right)},\cdots ,x^{\left(i\right)},\cdots ,x^{\left(m\right)}\right\}$ are the grayscale of *m* pixels in one picture which regarded as the input data. Among them, $x^{\left(i\right)}$ represents the grayscale of L*, a* or b* dimension of the *i*th pixel based on CIELAB color space. The specific steps to construct the color palette are as below.

Step I: Randomly select *j* cluster centroids $\left\{\mu_{1},\ldots ,\mu_{j}\right\}$ . Among them, *j* represents the number of cluster centroids, $\mu_{j}$ represents the grayscale of *R* channel, *G* channel, or *B* channel of the *j*th cluster centroids.

Step II: For each pixel $x^{\left(i\right)}$ , calculate the category that $x^{\left(i\right)}$ should belong to:


(9) $$c^{\left(i\right)}:\;=\;\;arg\min_{j}{\left\|x^{\left(i\right)}-\mu_{j}\right\|}^{2}$$

Among them, where $c^{\left(i\right)}$ is the target category with the shortest Euclidean distance between $x^{\left(i\right)}$ and all the *j* clustering centroids.

Step III: For each category $c^{\left(i\right)}$ , calculate the average value of $x^{\left(i\right)}$ which belongs to $c^{\left(i\right)}$ as the new cluster centroid $\mu_{j}$ .

Step IV: Repeat Step II and Step III until $\left\{\mu_{1},\ldots ,\mu_{j}\right\}$ remain unchanged, and $\left\{\mu_{1},\ldots ,\mu_{j}\right\}$ are the dominant colors of the multi-color material. $n_{j}$ represents the number of $x^{\left(i\right)}$ which belong to $c^{\left(i\right)}$ .

*Step V:* Generate the dominant colors based on the cluster centroids $\left\{\mu_{1},\ldots ,\mu_{j}\right\}$ , and calculate the length ratio of each dominant color based on $n_{j}$ , so as to construct the color palette.

**Figure 3:**
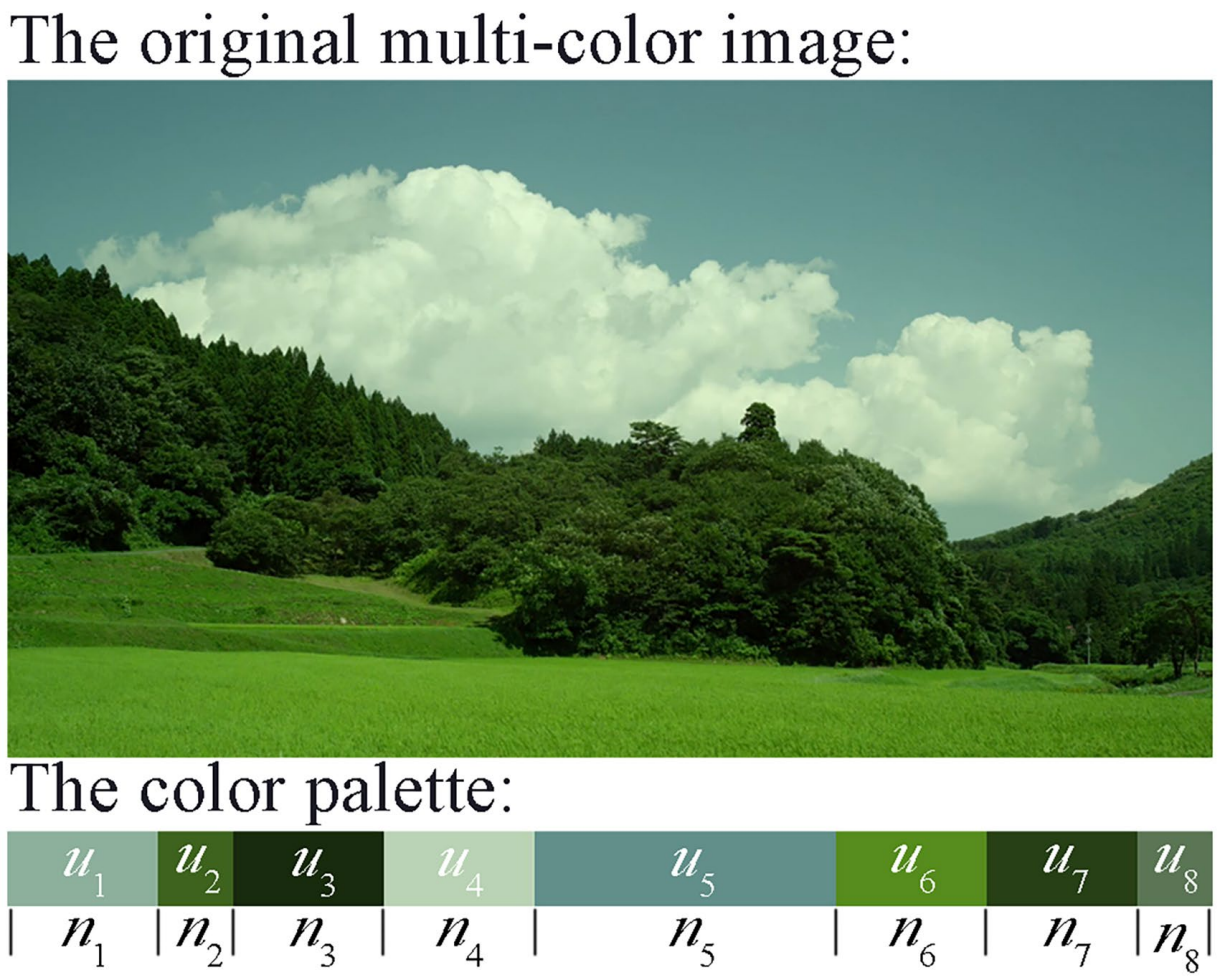
Color palette of a multi-color material generated by K-means clustering algorithm. Among them, $\mu_{j}$ represents each dominant color, and $n_{j}$ represents the length of each dominant color in the color palette. *j* is set to 8.

As shown in Figure 3, we generate a color palette to represent the dominant colors. In addition, it is worth noting that, there are three functions of the color palette. (1) Test whether the distribution of the hue attribute of multi-color materials is uniform and complete. (2) In the subjective evaluation of color harmony, the multi-color materials and the corresponding color palette will be presented at the same time, so as to make the experimental procedure more comprehensive and accurate. (3) Some multi-color physical features are extracted which taking the color palette as the input.

## Multi-color physical features extraction

Multi-color physical features can be directly quantified based on the existing color systems and the information technology methods, which follow the objective physical laws and have nothing to do with human’s cognition, also known as low-level features or objective features (Huang and Shen, 2002). In order to construct the multi-color harmony prediction model, this article first designs and quantifies 16-dimensioanl physical features of multiple colors, and then extracts features based on the smoothed multi-color materials, as shown in Table 1. The calculation method of each feature will be introduced in detail below.

**Table 1:** Sixteen-dimensional multi-color physical features.

No	Multi-color physical features	Abbreviation	Dimension
1	Color moment	CM	9
2	Color richness	CR	1
3	Space density	SD	2
4	Color tone contrast	CTC	1
5	lightness contrast	LC	1
6	Cool/warm contrast	CWC	1
7	area difference	AD	1

There are seven categories of multi-color physical features. From left to right, the second column represents the description of each feature, the third column represents the abbreviation composed of the capital letters from the corresponding feature description, and the fourth column represents the dimensions of each feature.

### Color moment

Color moment (Gonzalez and Woods, 2020) is a simple and effective physical feature to represent color, which mainly includes first-order moment, second-order moment, and third-order moment. Since the main information of color is distributed in the low-order moments, the first-order moment, the second-order moment, and the third-order moment are sufficient to express the color distribution of one multi-color material.

Based on CIELAB color space, this article quantified the first-order moment, the second-order moment, and the third-order moment of L*, a*, and b* dimensions to represent Color Moment (CM) feature, so as to construct a nine-dimensional feature vector.


(10) $$f_{1}=\frac{1}{N}\overset{N}{\underset{i=1}{\sum }}L^{*}{}_{i}$$

(11) $$f_{2}=\frac{1}{N}\overset{N}{\underset{i=1}{\sum }}a^{*}{}_{i}$$

(12) $$f_{3}=\frac{1}{N}\overset{N}{\underset{i=1}{\sum }}b^{*}{}_{i}$$

(13) $$f_{4}={\left[\frac{1}{N}\overset{N}{\underset{i=1}{\sum }}{\left(L^{*}{}_{i}-f_{1}\right)}^{2}\right]}^{1/2}$$

(14) $$f_{5}={\left[\frac{1}{N}\overset{N}{\underset{i=1}{\sum }}{\left(a^{*}{}_{i}-f_{2}\right)}^{2}\right]}^{1/2}$$

(15) $$f_{6}={\left[\frac{1}{N}\overset{N}{\underset{i=1}{\sum }}{\left(b^{*}{}_{i}-f_{3}\right)}^{2}\right]}^{1/2}$$

(16) $$f_{7}={\left[\frac{1}{N}\overset{N}{\underset{i=1}{\sum }}{\left(L^{*}{}_{i}-f_{1}\right)}^{3}\right]}^{1/3}$$

(17) $$f_{8}={\left[\frac{1}{N}\overset{N}{\underset{i=1}{\sum }}{\left(a^{*}{}_{i}-f_{2}\right)}^{3}\right]}^{1/3}$$

(18) $$f_{9}={\left[\frac{1}{N}\overset{N}{\underset{i=1}{\sum }}{\left(b^{*}{}_{i}-f_{3}\right)}^{3}\right]}^{1/3}$$

Among them, L*, a*, and b* represent the lightness value, red/green value, and yellow/blue value in CIELAB color space, respectively; *N* represents the number of pixels in each multi-color material; *i* represents the *i*th pixel.

### Color richness

Information entropy can be used to describe the chaotic degree of information, also called the degree of uncertainty, which is quantified by the occurrence probability of discrete random events (Li, 2008). The richer the color of a picture is, the greater the information entropy is. Therefore, this article constructed a one-dimensional feature vector by calculating the information entropy of the grayscale of each multi-color material grayscale to represent Color Richness (CR) feature.


(19) $$f_{10}=-\overset{255}{\underset{i=0}{\sum }}P\left(i\right)\cdot \log_{2}P\left(i\right)$$

Among them, P(i) represents the proportion of the pixels whose grayscale is *i* in one picture, when i∈[0,255] . That is, its unit grayscale entropy.

### Space density

The spatial density of multiple colors refers to how closely various colors are arranged in a picture, which has an impact on color harmony. In this article, the watershed segmentation algorithm (Beucher and Meyer, 1993; Hu, 2010; Shen et al., 2015) based on region segmentation is adopted to segment the color regions in multi-color materials.

The watershed segmentation algorithm is a segmentation method that draws on the morphological theory, which takes the picture as a topographic map, where the grayscale f(x,y) corresponds to the topographic height value. High grayscale corresponds to peaks, and low grayscale corresponds to valleys. The water always flows toward the low ground and stops until a low-lying place, which is called a basin. Eventually, all the water will be concentrated in different basins, and the ridges between the basins are called watersheds. As water flows down from the watershed, it is equally likely to flow toward different basins.

Applying this idea to image segmentation is to find different “water basins” and “watersheds” in grayscale images, and the regions composed of these different “water basins” and “watersheds” are the target to be segmented. The bottom-up simulated flooding process is defined as follows.


(20) $$X_{h_{\min}}=T_{h_{\min}}\left(I\right),\forall h\in \left[h_{\min},h_{\max}-1\right]$$

(21) $$X_{h+1}=\min_{k+1}\cup C_{X_{h}}\left(X_{h}\cap X_{h+1}\right)$$

Among them, Equation (20) belongs to the initial condition of the recursive process. Among them, $T_{h_{\min}}\left(I\right)$ is the pixel point with the minimum grayscale in image I; *h* represents the range of grayscales; $h_{\min}$ represents the minimum value; $\mathrm{and}\;h_{\max}$ represents the maximum value. Equation (21) is the recursive process. Among them, $X_{h+1}$ represents all the pixels which grayscale is h + 1; $\min_{k+1}$ the pixel with the minimum gray scaled which belongs to the newly generated basin, that is, a new basin is generated at the altitude of *h + 1*. $X_{h}\cap X_{h+1}$ represents the intersection point of $X_{h}$ and $X_{h+1}$ ; $C_{X_{h}}$ represents the basin where $X_{h}$ is located. Therefore, $X_{h}\cap X_{h+1}$ also represents the point where $X_{h}$ and $X_{h+1}$ are in the same basin $C_{X_{h}}$ .

Based on the recursive process, all the pixels in the image I can be divided into several basins. Finally, if a pixel belongs to more than two basins at the same time, the pixel is a point on the watershed. The segmentation line can be further determined by the points on the watershed, thereby realizing image segmentation.

On the basis of regional segmentation of multi-color materials, this article quantifies the number of regions segmented and the standard deviation of the number of pixels contained in each region in the same material as the Space Density (SD) feature to construct a two-dimensional feature vector.


(22) $$f_{11}=N$$

(23) $$f_{12}=\sqrt{\frac{1}{N}\overset{N}{\underset{i=1}{\sum }}{\left(n_{i}-\bar{n}\right)}^{2}}$$

Among them, *N* represents the number of segmented regions in one material; $n_{i}$ represents the number of pixels included in the *i*th segmented region in one material.

### Color tone contrast

Since color tones contain information both on the hue and chroma, this article selects CIELAB color space to quantify this feature, which well separated the lightness property from the color tone property. A one-dimensional feature vector was constructed by calculating the standard deviation of a* and b* of each pixel in one material to represent the Color Tone Contrast (CTC) feature.


(24) $$f_{13}={\left[\frac{1}{N}\overset{N}{\underset{1}{\sum }}\left({\left(a_{i}^{*}-\bar{a^{*}}\right)}^{2}+{\left(b_{i}^{*}-\bar{b^{*}}\right)}^{2}\right)\right]}^{1/2}$$

Among them, $a_{i}^{*}$ and $b_{i}^{*}$ represent $a_{i}^{*}$ and $b_{i}^{*}$ of the *i*th pixel of one material in CIELAB color space respectively; $\bar{a^{*}}$ and $\bar{b^{*}}$ represent the average values of a* and b*; and *N* represents the number of pixels in one material.

### Lightness contrast

Based on CIELAB color space, this article takes the lightness value of L* of the pixel (i,j) in each material as the input to quantify the Lightness Contrast (LC) feature constructed by the one-dimensional feature vector.


(25) $$f_{14}=\frac{\sum_{\left(i,j\right)}\sum_{\left(x,y\right)\in \Omega }{\left|L^{*}\left(i,j\right)-L^{*}\left(x,y\right)\right|}^{2}}{N}$$

**Figure 4:**
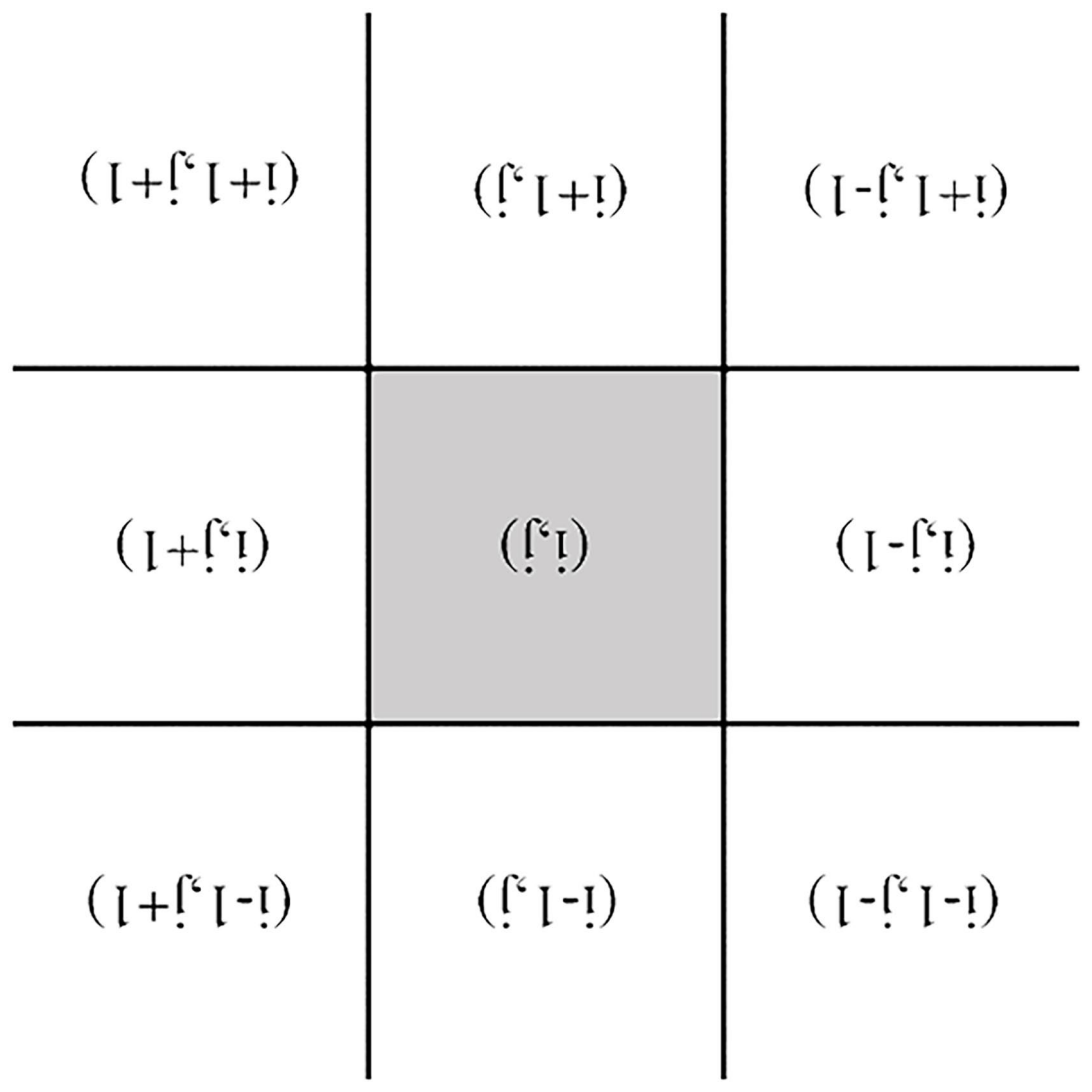
The image block Ω of eight neighborhoods that center on the pixel ( (i,j) . (x,y)∈Ω ).

Among them, the pixel (x,y) represents the pixel within the block Ω of eight neighborhoods that center on the pixel (i,j) , as shown in Figure 4. *N* represents the number of pixels different from pixel (i,j) .

### Cool/warm contrast

Due to the strong correlation between the cool/warm and multicolor perception, this article defined and quantified the Cool/warm Contrast (CWC) feature based on the earlier research of Ou et al. (2018). Based on CIELAB color space, the hue angle and chroma values of the pixel (i,j) in one material is adopted as the input, and the steps are below.

Step I: Calculate the hue angle *h* and chroma C* values based on CIELAB color space.


(26) $$h_{i}=\tan^{-1}\left(\frac{b_{i}^{*}}{a_{i}^{*}}\right)$$

(27) $$C_{i}^{*}=\sqrt{{\left(a_{i}^{*}\right)}^{2}+{\left(b_{i}^{*}\right)}^{2}}$$

Among them, $a_{i}^{*}$ and $b_{i}^{*}$ represent $a_{i}^{*}$ and $b_{i}^{*}$ of the *i*th pixel of one material in CIELAB color space, respectively.

Step II: Calculate the degree of “cool/warm” based on the research result of Ou.


(28) $$\begin{array}{l} WC_{i}=-0.89+0.052C_{i}^{*}[\cos(h_{i}-50{}^{\circ})+0.16\cos\\ \left(2h_{i}-350{}^{\circ})\right] \end{array}$$

Among them, $a_{i}^{*}$ and $b_{i}^{*}$ represent $a_{i}^{*}$ and $b_{i}^{*}$ of the *i*th pixel of one material in CIELAB color space, respectively.

*Step III:* Calculate the Cool/warm Contrast (CWC) feature based on $WC_{i}$ .


(29) $$f_{15}=\frac{\sum_{\left(i,j\right)}\sum_{\left(x,y\right)\in \Omega }{\left|WC\left(i,j\right)-WC\left(x,y\right)\right|}^{2}}{N}$$

Among them, the pixel (x,y) represents the pixel within the block Ω of eight neighborhoods that center on the pixel (i,j) ; *N* represents the number of pixels different from pixel (i,j) .

### Area difference

The importance of area in color design has been proved in the field of aesthetics and art practice, and researchers have also conducted a series of studies on the mechanism of area on color harmony and beauty (Granger, 1953; Odabaşıoğlu and Olguntürk, 2020). Therefore, the standard deviation of the number of pixels $n_{j}$ belonging to each cluster centroid $\mu_{j}$ in the color palette was quantified to represent the Area Difference (AD) feature, thereby constructing a one-dimensional feature vector.


(30) $$f_{16}={\left[\frac{1}{j}\overset{j}{\underset{i=1}{\sum }}{\left(n_{i}-\bar{n}\right)}^{2}\right]}^{1/2}$$

Among them, *j* represents the number of cluster centroids; $n_{i}$ represents the number of pixels belonging to the *i*th cluster centroid.

## The subjective evaluation experiment on color harmony

### Subjects

A total of 84 subjects participated in this experiment. The subjects are all college students, aged between 18 and 30 years old, including 35 males and 49 females. All the subjects do not major in color-related professional courses and have no color-related professional knowledge reserves. Before the formal start of the experiment, the Ishihara Color Blindness Test (Marey et al., 2015) is performed on each subject to test their color perception.

### Experiment condition

In order to avoid the interference of environmental noise on the subjective evaluation of multi-color harmony, the experiment is arranged in a standard listening room with an area of 5.37 m × 6 m to ensure that the background noise is not higher than 30 dB(A). According to “Methodology for the Subjective Assessment of the Quality of Television Pictures” (ITU-R BT. 500–14), the ambient illumination of the monitor (i.e., the incident light formed by the surrounding environment on the monitor, measured in the vertical direction of the monitor) is set to 200 lux (a unit of luminance, which represents the amount of light received per unit area of the monitor).

An independent monitor is used to present multi-color materials. The model of the monitor is BenQ XL2720-B color LCD monitor, equipped with Windows operating system (7, Microsoft Corporation, Redmond, United States). The aspect ratio of the monitor is 16:9, with the resolution of 1920 dpi × 1,080 dpi. A “slideshow” function in the ACD see software (official free version; ACD Systems International Inc., Shanghai, China) is used to randomly present the stimuli. Before the experiment, the monitor is calibrated using the Display Color Calibration Function from Windows Operating System before the experiment.

### Experiment procedure

Only one subject performs the experiment at a time. After the subject enters into the laboratory, a questionnaire is issued by the experimental assistant. The subject is required to sign the informed consent form and fill in the personal information (including gender, age, and major) in the questionnaire.

**Figure 5:**
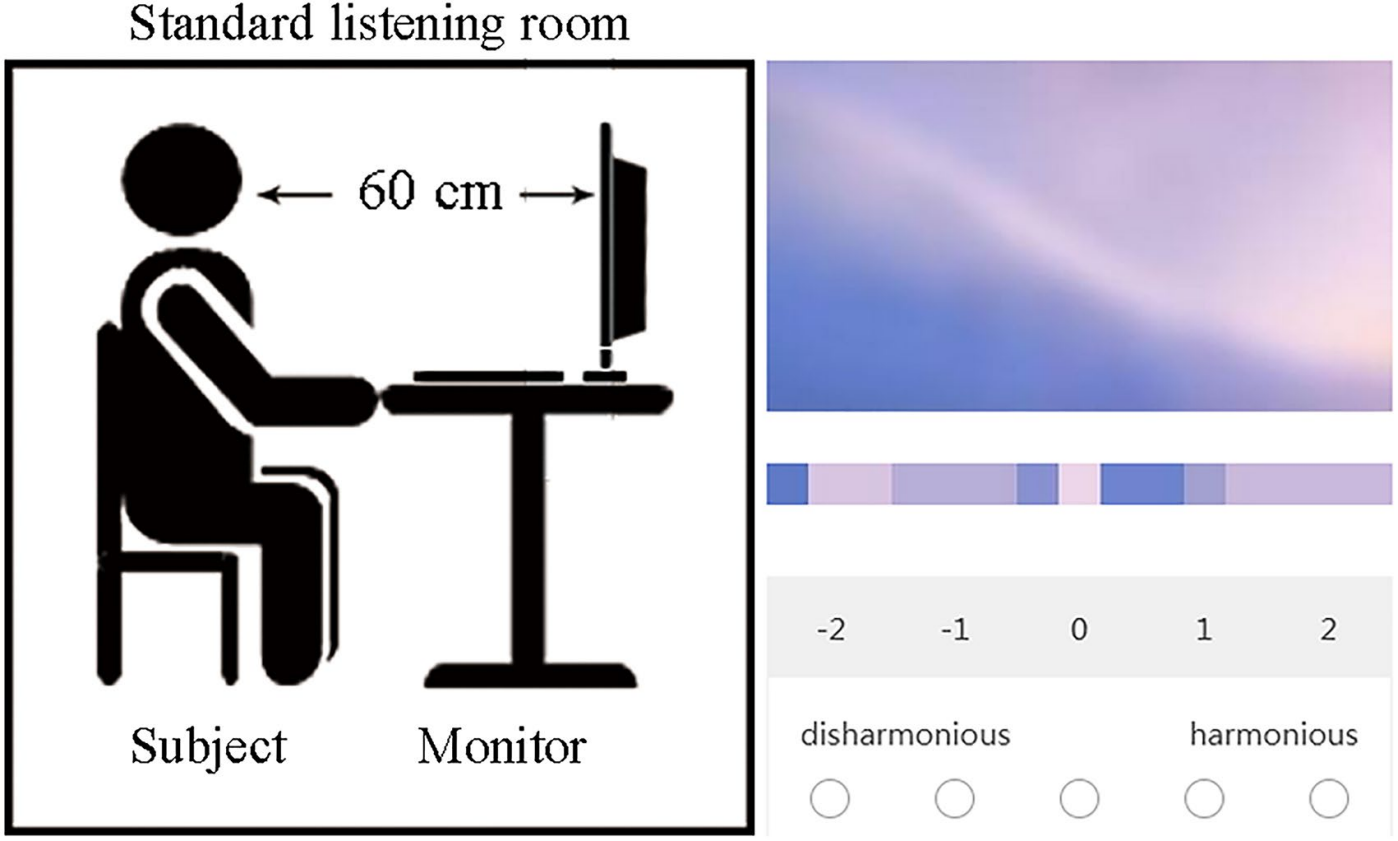
The experiment condition. On the left is the position diagram between the subject and the monitor; on the right is the operation interface for data collection by “wjx.cn” application on the mobile phone.

Then, as shown in Figure 5, the subject is asked to sit in front of the monitor with the eyes at the same height as the center of the monitor, and the vertical distance between the eyes and the monitor is 60 cm. The monitor randomly displays each material, including the smoothed multi-color material and the corresponding color palette, to be evaluated in full screen, and the color harmony scores are collected through the “wjx.com” application of the mobile phone.

The introduction of the experiment is below, “The aim of this experiment is to study the quantifiable rules of color harmony. You should score a total of 164 multi-color materials for the degree of color harmony as a 5-level scale, which −2 means “disharmonious,” −1 means “a little disharmonious,” 0 means “no feeling,” 1 means “a little harmonious,” and 2 means “harmonious.” The detailed experiment is below.

Step I: The subject is asked to be familiar with all multi-color materials in advance, and constructs a psychological evaluation scale on color.

Step II: Three materials randomly selected are displayed, and the subject is asked to evaluate according to his subjective feelings, so as to avoid the effect on the evaluation results because of the unfamiliarity with the experimental process.

Step III: In total, 164 multi-color materials are equally divided into two groups and present on the monitor in random order. The subject is asked to score the degree of each multi-color material as a 5-level scale.

It is noted that there is no time limit for the experiment, and the subjects can play the next material by themselves. It took 1 week to complete the experiments with all 84 subjects. Among them, each participant spent an average of about 30 min in the experiment, including a 5-min break after evaluating one group.

## Results and discussion

First, in section “Reliability analysis,” this section analyzes the reliability of the experimental data. Second, in section “Correlation analysis,” the correlation between each multi-color physical feature and color harmony is discussed in detail. Then, in section “Multi-color harmony model construction,” based on the results of feature selection, linear and nonlinear learning algorithms are adopted to construct the regression prediction model and classification prediction model for color harmony, and a series of objective evaluation methods are used to measure the accuracy of the models. Finally, in section “Comparative research”, a comparative experiment is carried out to discuss the applicability and limitations of the proposed models.

### Reliability analysis

In this article, Cronbach’s alpha is adopted to evaluate the internal consistency of the experimental results of 84 subjects (Bartko, 1966), as shown in Equation (31).


(31) $$\alpha =\frac{K}{K-1}\left(1-\frac{\sum_{i=1}^{K}\sigma_{Y_{i}}^{2}}{\sigma_{X}^{2}}\right)$$

Among them, *K* represents the number of subjects; $\sigma_{X}^{2}$ represents the population variance for the observation scores of all the materials; $\sigma_{Y_{i}}^{2}$ represents the variance for the observation scores of the material to be tested. It is generally believed that when the Cronbach’s alpha is above 0.7, the experimental results have good internal consistency.

The Cronbach’s alpha of the 84 subjects in this experiment is 0.908, which meets the requirement, indicating that the experimental data are reliable and can support subsequent research on correlation analysis and modeling.

### Correlation analysis

**Figure 6:**
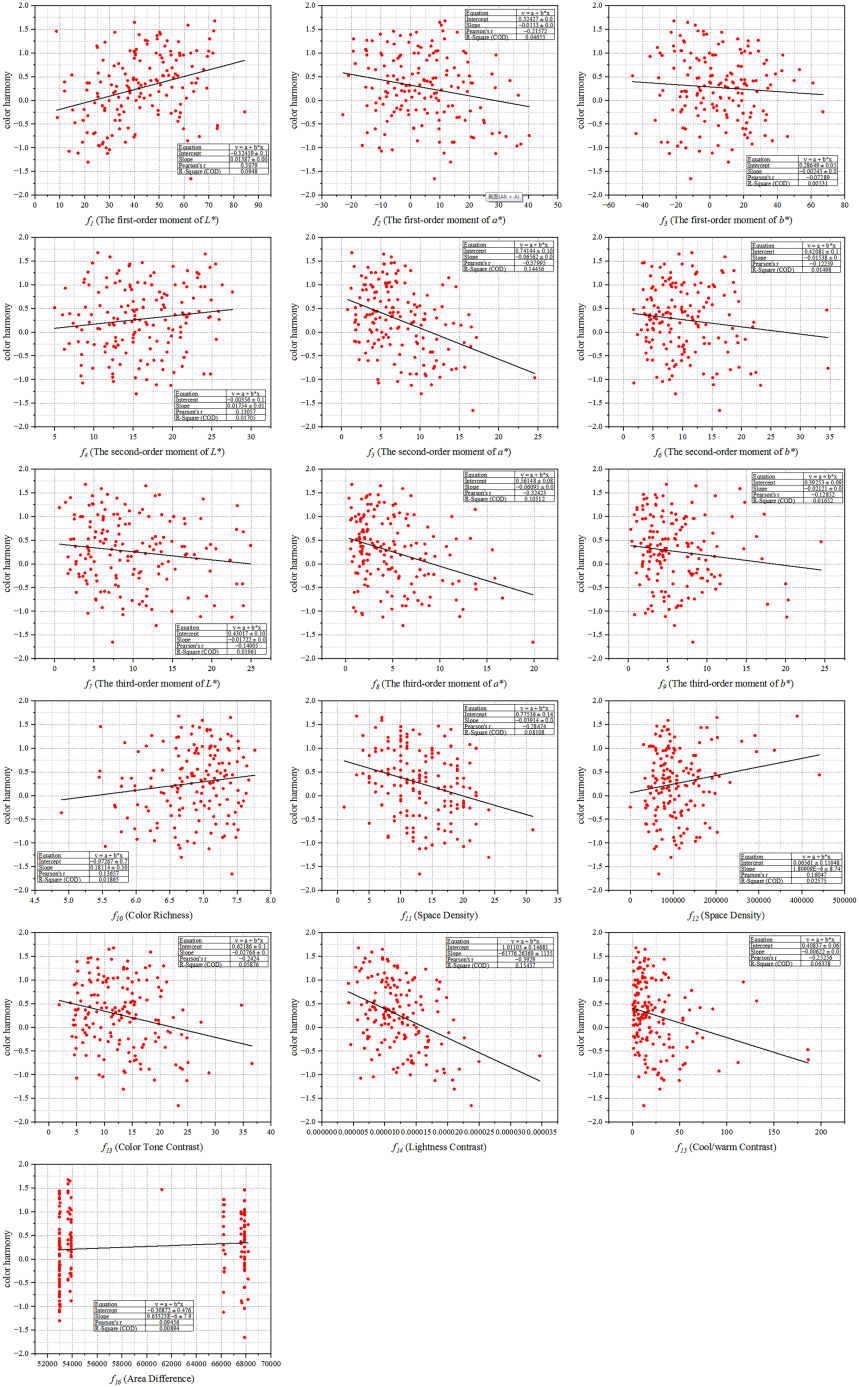
The scatter plots between multi-color physical features and color harmony. Among them, *X*-axis represents each multi-color physical feature, and *Y*-axis represents the corresponding value of the degree of color harmony.

After acquiring the data of multi-color physical features and the subjective evaluation scores on color harmony of all multi-color materials, it is necessary to draw the scatter plots and fitting curves to conduct a qualitative analysis of the relationship between multi-color physical features and color harmony, and then make a quantitative analysis through the correlation coefficient. Figure 6 shows the scatter plots between multi-color physical features and color harmony.

It can be seen from Figure 6 that some multi-color physical features have weak correlation with color harmony, namely $f_{1}$ (The first-order moment of L*), $f_{2}$ (The first-order moment of a*), $f_{5}$ (The second-order moment of a*), $f_{8}$ (The third-order moment of a*), $f_{11}$ (Space Density), $f_{13}$ (Color Tone Contrast), $f_{14}$ (Lightness Contrast), and $f_{15}$ (Cool/warm Contrast). Among them, the multi-color physical feature $f_{1}$ (The first-order moment of L*) is positively correlated with color harmony; the multi-color physical features $f_{2}$ (The first-order moment of a*), $f_{5}$ (The second-order moment of a*), $f_{8}$ (The third-order moment of a*), $f_{11}$ (Space Density), $f_{13}$ (Color Tone Contrast), $f_{14}$ (Lightness Contrast), and $f_{15}$ (Cool/warm Contrast) are negatively correlated with color harmony. That is to say, the brighter, cooler, simpler, and smaller the difference among multiple colors is, the multi-color relationship is more likely to make people feel harmonious.

Then, the Pearson’s correlation coefficients ( r ) and fitting curves are also presented in scatterplots. It is generally believed that if 0.7≤|r| , it has a strong correlation; if 0.5≤|r|<0.7 , it has a moderate correlation; if 0.2≤|r|<0.5 , it has a weak correlation; and if |r|<0.2 , there is no correlation. The Pearson’s correlation coefficient of $f_{14}$ (Lightness Contrast) is the highest, which is: r=−0.393,p<0.001 . In addition, the multi-color physical features $f_{1}$ (The first-order moment of L*), $f_{5}$ (The second-order moment of a*), and $f_{8}$ (The third-order moment of a*) are also correlated with color harmony, with the Pearson’s correlation coefficients being r=0.308,p<0.001 , r=−0.38,p<0.001 , and r=−0.324,p<0.001 . Finally, the multi-color physical features $f_{2}$ (The first-order moment of a*), $f_{11}$ (Space Density), $f_{13}$ (Color Tone Contrast), and $f_{15}$ (Cool/warm Contrast) have weak correlation with color harmony, with the Pearson’s correlation coefficients being r=−0.216,p<0.001 , r=−0.285,p<0.001 , r=−0.242,p<0.001 , and r=−0.253,p<0.001 .

To sum up, the lower the lightness contrast of the multi-color combination, the easier it is to make people feel harmonious; the higher the lightness of the multi-color combination, the easier it is to make people feel harmonious; the cooler the multi-color combination, the smaller the difference among multiple colors, the lower the spatial density of the color blocks, the lower the color tone contrast, the lower the cool/warm contrast, and the easier it is to make people feel harmonious.

### Multi-color harmony model construction

In order to further study the correlation between multi-color physical features and color harmony, machine learning algorithms are adopted to construct the multi-color harmony prediction model on the basis of correlation analysis. In order to eliminate the dimensional difference between different multi-color physical features, so as to further improve the accuracy of the prediction model. This article first normalizes the input multi-color physical eigenvalue $x_{i}.$


(32) $$x_{i}=\frac{x_{i}-\min\left\{x\right\}}{\max\left\{x\right\}-\min\left\{x\right\}}$$

Among them, min{x} represents the minimum of the eigenvector *X*; max{x} represents the maximum of the eigenvector *X*.

#### Linear regression predication model

The Multivariable Linear Regression (MLR) (Hidalgo and Goodman, 2013) has the characteristics of strong interpretability. Therefore, MLR is adopted to construct the regression predication model. Assume that there is a linear relationship between the independent variables $X_{1},X_{2},\ldots ,⊃X_{p}$ and the dependent variable y , as shown in Equation (25).


(33) $$y=\beta_{0}+\beta_{1}X_{1}+\ldots +\beta_{p}X_{p}+\varepsilon$$

Among them, $\beta_{0},\beta_{1},\;\ldots ,\;\;\beta_{p}$ represents the unknown parameters, $\beta_{0}$ is the regression constant, and $\beta_{1},\beta_{2},\;\ldots ,\;\;\beta_{p}$ represents the overall regression parameters; ε is a random error which obeys the $\varepsilon \sim N\left(0,\sigma^{2}\right)$ distribution. When p=1 , the equation is called the univariate linear regression model; when p≥2 , the equation is called the MLR model. The Ordinary Least Square (OLS) method is adopted to estimate the overall regression parameters $\beta_{0},\beta_{1},\;\ldots ,\;\;\beta_{p}$ , and calculate the value of β to minimize the object function.


(34) $$Q\left(\beta \right)=\overset{n}{\underset{i=1}{\sum }}||y_{i}-x_{i}\beta |{|}^{2}$$

In this article, the 10-fold Cross Validation is used to evaluate the accuracy of the model, that is, the dataset is divided into 10 subsets, and nine of them are taken as training sets without repetition, and the prediction errors of the remaining subsets are evaluated. And the evaluation indexes are the Pearson’s correlation coefficient ( r ), Mean Absolute Error (MAE), and Root Mean Squared Error (RMSE), respectively. The calculation methods of MAE and RMSE are below.


(35) $$MAE=\frac{1}{n}\overset{n}{\underset{i=1}{\sum }}\left|y_{i}-f\left(x_{i}\right)\right|$$

(36) $$RMSE=\sqrt{\frac{1}{n}\overset{n}{\underset{i=1}{\sum }}{\left(y_{i}-f\left(x_{i}\right)\right)}^{2}}$$

Among them, $f\left(x_{i}\right)$ represents the prediction value; $y_{i}$ represents the true value; n represents the number of material in testing set.

The weka software (3.8.3; The University of Waikato, Hamilton, New Zealand) with functions of machine learning and data mining was adopted to construct the MLR model. In addition, we apply the M5 rules (Duggal and Singh, 2012) as the attribute selection method, that is, adding the classification discriminators before the linear regression model, so as to construct the piecewise linear regression model to improve the accuracy. Finally, based on the M5 rules, the linear regression prediction model is constructed as follows.


(37) $$\begin{array}{l} colorharmony=1.499f_{1}+0.408f_{4}-0.594f_{5}\\ -0.52f_{11}-0.47f_{13}-1.386f_{14}+0.862f_{15}+0.249 \end{array}$$

Among them, $f_{1}$ represents the first-order moment of L*; $f_{4}$ represents the second-order moment of L*; $f_{5}$ represents the second-order moment of a*; $f_{11}$ represents space density; $f_{13}$ represents color tone contrast; $f_{14}$ represents lightness contrast; $and\;f_{15}$ represents cool/warm contrast.

#### Non-linear regression predication model

**Figure 7:**
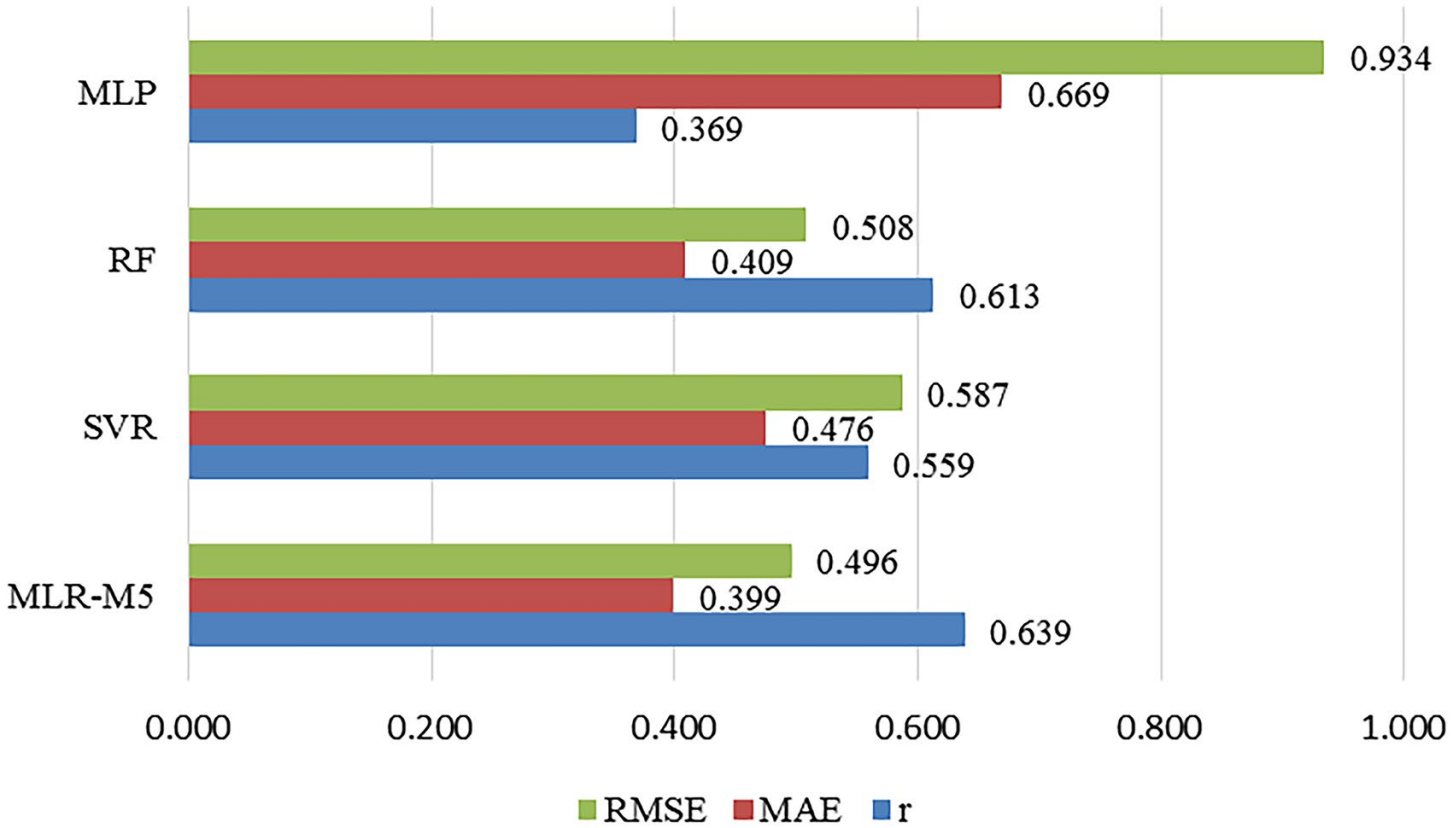
The comparison of the regression prediction results of the five machine learning algorithms.

On the basis of constructing the linear regression models, this article tries to apply nonlinear machine learning algorithms into regression model construction, so as to further explore the relationship between multi-color physical features and color harmony. The three classic nonlinear machine learning algorithms used in this article are the Support Vector Regression (SVR), Random Forest (RF), and Multi-layer Perception (MLP). The comparison of the prediction results of the four machine learning algorithms is shown in Figure 7.

It can be seen that the accuracy of the linear regression model is higher than those of the nonlinear regression models, with the prediction accuracy of MLR being 63.9%. Therefore, the relationship between the multi-color physical features and color harmony designed and quantified in this article can be constructed through the linear regression model, so as to give a reasonable explanation for the influencing factors of color harmony. On the other hand, among the nonlinear machine learning algorithms, the prediction accuracy of SVR and RF are close, which are 55.9% and 61.3%, respectively, and the prediction performance of RF is slightly better than that of SVR. In addition, the prediction accuracy of MLP is poor, which is 36.9% only.

#### Classification predication model

Since the prediction accuracy of the regression model of color harmony needs to be improved, this article attempts to transform the problem into a binary classification problem, and machine learning algorithms are also adopted to construct the classification prediction model. Set the classification threshold T=0 , that is, the degree of color harmony greater than 0 belongs to the “harmonious” category, and the degree of color harmony less than 0 belongs to the “disharmonious” category. The Logical Classification (LC) (Povhan, 2020), Support Vector Machine (SVM) (Ohata et al., 2021), RF (Lei et al., 2022), and MLP are, respectively, adopted to construct the classification prediction models (Gao et al., 2019). In the modeling process, 10-fold Cross Validation is used to better optimize the model parameters during the modeling process and reduce the evaluation errors. In terms of evaluation methods, this article measures the prediction accuracy of the classification model by the Correct Classification Rate (CC), MAE, and RMSE. The CC rate calculation method is as below.


(38) CC=(TP+TN)(P+N)

**Figure 8:**
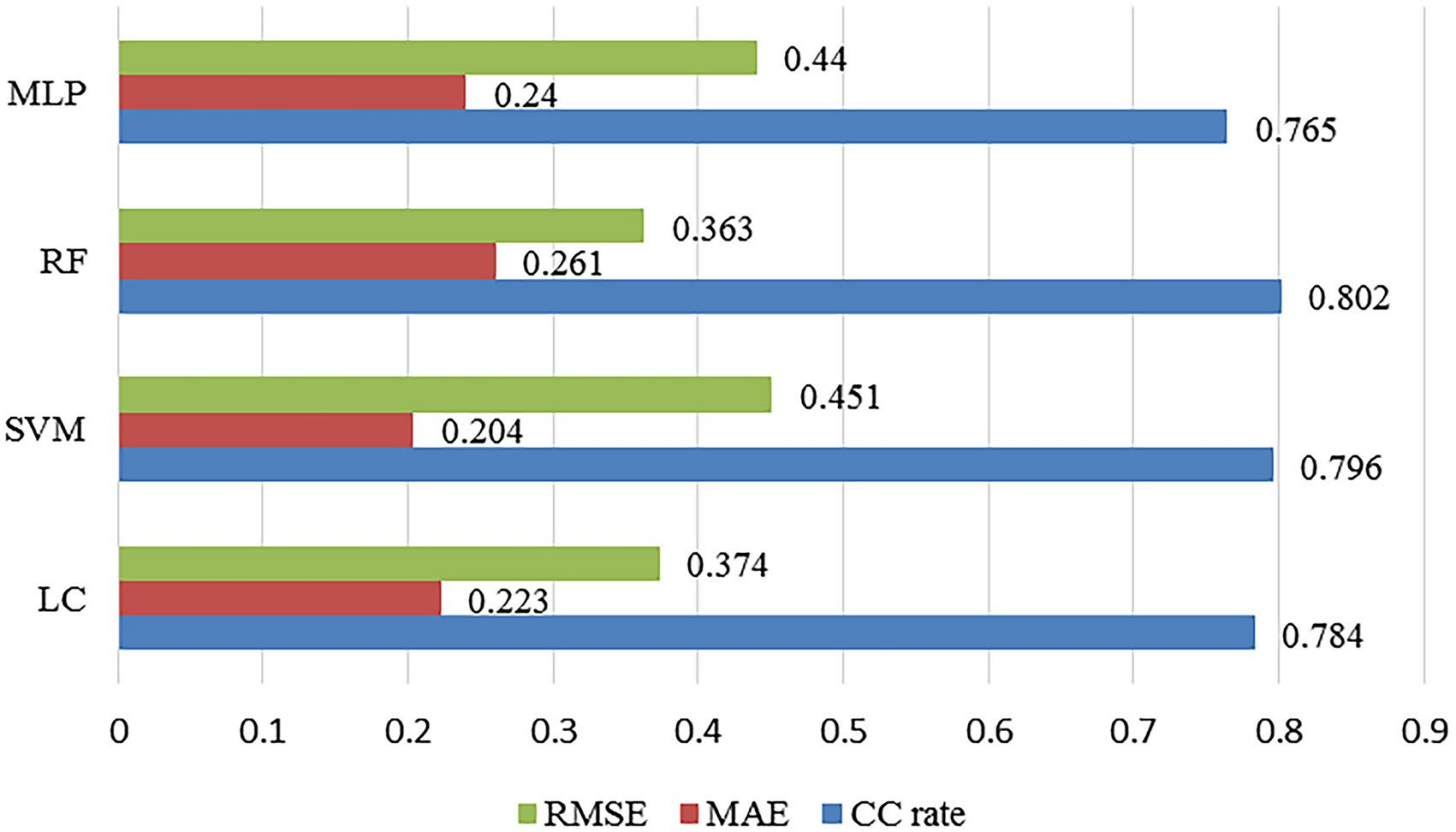
The comparison of the classification prediction results of the four machine learning algorithms.

Among them, *TP* represents the number of the true samples predicted as positive by the model; *TN* represents the number of the true samples predicted as negative by the model; *P* represents the number of the true samples; and *N* represents the number of the false samples. The comparison of the prediction results of the four machine learning algorithms is shown in Figure 8.

It can be seen that the overall performance of the classification prediction models constructed in this article plays better than that of the regression models. Among them, RF has the best classification prediction performance, with the classification accuracy rate of 80.2%. In addition, LC and SVM have similar classification accuracy rates, which are 78.4 and 79.6%, respectively. And MLP also has the poorest classification prediction performance, with the classification accuracy of 76.5%.

### Comparative research

#### Comparison with other datasets

**Figure 9:**
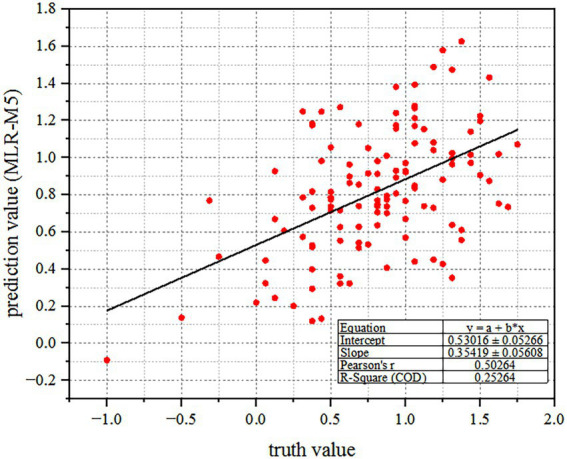
The scatterplot between the truth value and the prediction value. Among them, *X*-axis represents the truth value of color harmony, and *Y*-axis represents the corresponding prediction value by the proposed regression model.

In order to verify the applicability of the proposed regression predication models of color harmony, we construct a dataset containing 120 new screenshots of film and television with the 5-scale color harmony scoring. Then, after 16-dimensionl multi-color physical features extracted, the Equation (37) by MLR with the M5 rules is adopted to predict color harmony of the new dataset. And the prediction accuracy evaluated by the Pearson’s correlation coefficients ( r ) is up to 50.3%, indicating that the proposed model has a good performance on the similar dataset. The scatterplot and fitting curve between the truth value and the prediction value is shown in Figure 9.

On this basis, in order to further test the prediction performance of the proposed regression model on other dissimilar datasets, we select one public dataset for comparative research: the *International Affective Picture System (IAPS)* (Lang et al., 2008). IAPS is the most widely used dataset for experimental investigations of emotion and attention. It consists of documentary-style natural color photos depicting scenes labeled by pleasure (*P*), arousal (*A*), and dominance (*D*) based on PAD emotion space (Mehrabian, 1980; Russel, 1980). Then, the calculation method of color harmony is below based on the research on the relationship between color harmony (CH) and emotion from Wang et al. (2022). A total of 90 materials are selected in our comparative experiment on the proposed regression prediction model with the obvious semantic information, e.g., people with clear expressions, excluded.


(39) CH=0.813P−0.616A+0.374

**Figure 10:**
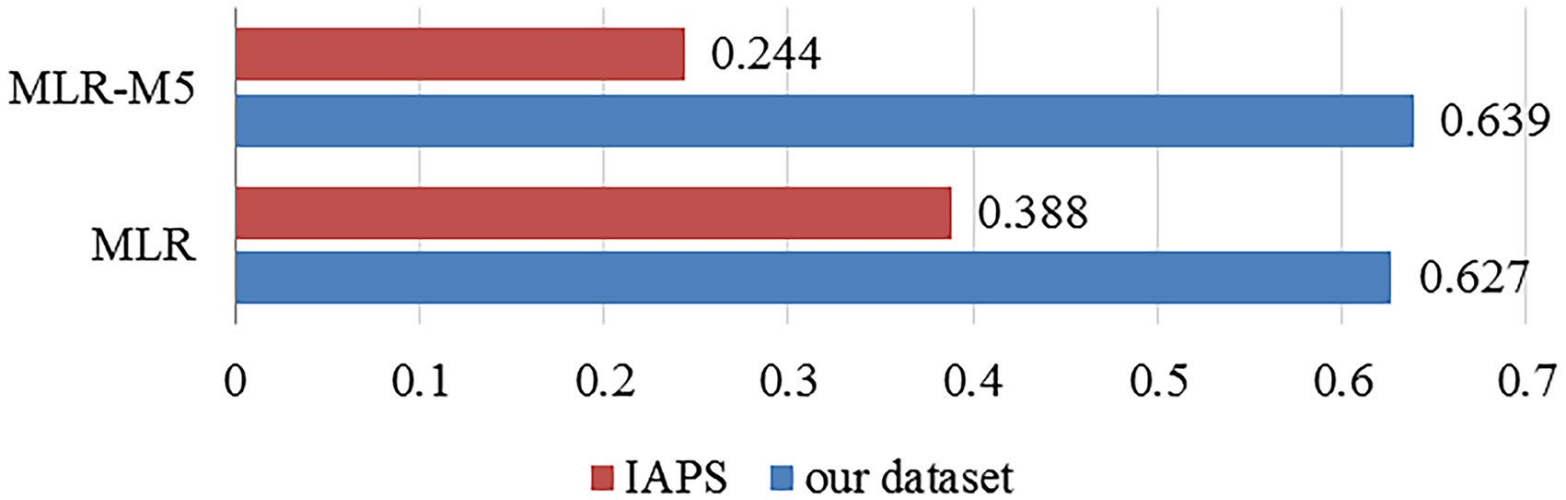
The comparison of the regression prediction results of the different datasets.

We try to use Equation (37) by MLR with/without the M5 rules to predict color harmony. And the Pearson’s correlation coefficients ( r ) between the truth values and the prediction values are r=0.388,p<0.001 and r=0.244,p<0.001 , respectively. Figure 10 shows the comparison of the regression prediction results of the different datasets.

It is noted that the accuracy of IAPS is lower than that of the screenshots of film and television scenes constructed by ourselves, the possible reasons are as follows. (1) The dataset in this article consists of the screenshots of film and television with color design, and the resolution of each material is high with the same format, to avoid the effect on data labeling. (2) Compared with the subjects in this article, the subjects for IAPS have a wider age range. Previous studies (Zhang et al., 2019) have shown that people with different ages have different color preferences, which may affect the judgment for color harmony. Therefore, the prediction models constructed in this article are more suitable for the field of art and design, where the pictures to be analyzed are of high quality.

In addition, different from the prediction performance on our dataset, the MLR model without the M5 rules is more accurate than that with the M5 rules on IAPS, indicating the overfitting problem.

#### Comparison with different sample sizes

**Figure 11:**
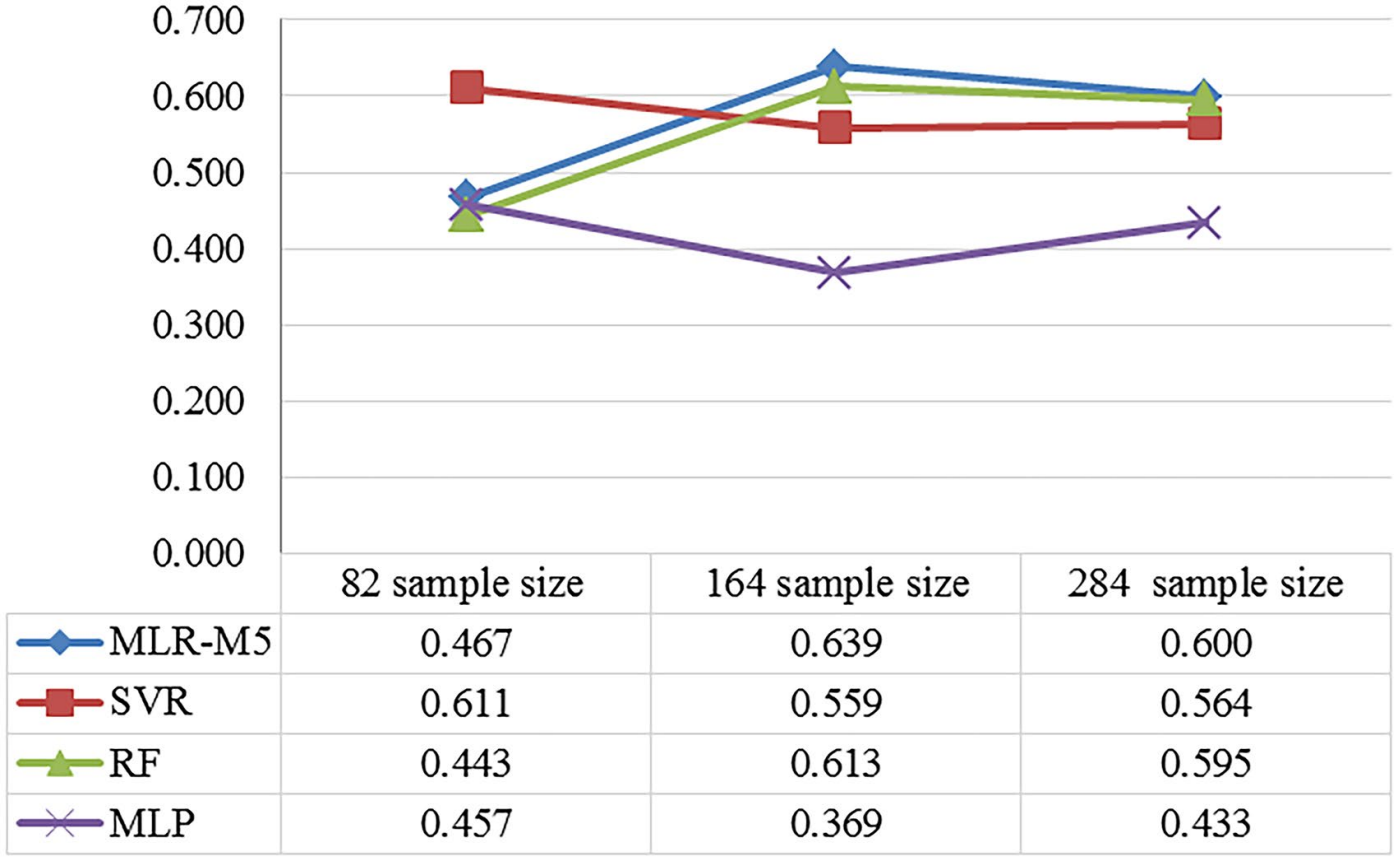
The comparison of the regression prediction results of the different sample sizes.

Since the accuracy of the regression models is not very ideal except for MLR, we try to use different sample sizes to train the model, so as to analyze the influence of sample size on the experimental results. Based on the dataset we constructed in the article, we add 120 screenshots of film and television with the 5-scale color harmony scoring (constructed in section “Comparison with other datasets”). Then, we conduct the comparative experiment on 82, 164, and 284 sample sizes which were selected randomly, respectively. The comparative result measured by the Pearson’s correlation coefficient ( r ) is shown in Figure 11.

It can be seen that the average r values of the 82, 164, and 284 sample sizes are 49.4, 54.5, and 54.8%, respectively, which grows with the sample size. In addition, the average r values of MLR, SVR, RF, and MLP are 56.8, 57.8, 55, and 42%, respectively. Then, for the explainable MLR, the 164 sample size has the highest prediction accuracy. To sum up, when the sample size is small, it negatively affects the accuracy of the predictive model; when the sample size reaches a certain size, it is beneficial to improve the accuracy of the prediction model through strict subjective experimental labeling, which provides the idea for few-shot learning. In addition, if the extracted physical features have strong correlation with the subjective ground truth, it is beneficial to construct the explainable linear models.

## Conclusion

In this article, we combine experimental psychology and information technology together to study the mechanism of color harmony in real-life scenes. The main contribution is attributed analysis and model construction of color harmony. Especially, (1) Design and quantify seven categories, 16-dimensional, multi-color physical features which are suitable for extracting in real-life scenes, namely Color Moment, Color Richness, Space Density, Lightness Contrast, Color Tone Contrast, Cool/Warm Contrast, and Area Difference. The correlation analysis shows that the overall lightness, difference of the color tones, number of multiple colors, lightness contrast, color tone contrast, and cool/warm contrast are significantly related to color harmony. (2) Based on the machine learning algorithms, the color harmony regression prediction model and the classification prediction model are, respectively, constructed. Among them, the prediction accuracy of the linear regression model is higher than that of the nonlinear regression model, with a maximum of 63.9%, indicating that the multi-color physical features designed and quantified in this article are effective and color harmony can be correlated through the linear regression model, so as to give reasonable explanations for the mechanism of color harmony. For the classification models, RF has the best prediction performance, with a prediction accuracy of 80.2%.

Future works are below. (1) Taking the classical color harmony theories into consideration, design and quantify the physical features of multiple colors that are consistent with the theoretical description, so as to fully explore the influencing factors of color harmony. (2) Previous studies have shown that (Jonauskaite et al., 2020), color preference is easily affected by gender, age, and cultural background. Therefore, we plan to introduce personalized factors as constraints into the prediction model by supplementing the dataset and increasing the number of differentiated subjects to further improve the prediction accuracy of the model. (3) For industry applications, develop color-matching software to increase the practicability of research results.

## Data availability statement

The original contributions presented in the study are included in the article/Supplementary material, further inquiries can be directed to the corresponding author.

## Ethics statement

Ethical review and approval was not required for the study on human participants in accordance with the local legislation and institutional requirements. The patients/participants provided their written informed consent to participate in this study.

## Author contributions

SW and YJ constructed the overall research framework. SW designed and quantified multi-color physical features and revised the manuscript. JLa and JJ constructed the material dataset. JLi carried out the subjective evaluation experiment. SW and JLi completed the correlation analysis and model construction and drafted the manuscript. All authors contributed to the article and approved the submitted version.

## Funding

Funding was provided by the National Key Research and Development Program (Nos. 2021YFF0901705 and 2021YFF0901700) and Open Project of Key Laboratory of Audio and Video Restoration and Evaluation, Mini of Culture and Tourism (2021KFKT006).

## Conflict of interest

JJ is employed by China Digital Culture Group Co., Ltd.

The remaining authors declare that the research was conducted in the absence of any commercial or financial relationships that could be construed as a potential conflict of interest.

## Publisher’s note

All claims expressed in this article are solely those of the authors and do not necessarily represent those of their affiliated organizations, or those of the publisher, the editors and the reviewers. Any product that may be evaluated in this article, or claim that may be made by its manufacturer, is not guaranteed or endorsed by the publisher.

## Supplementary material

The Supplementary material for this article can be found online at: https://www.frontiersin.org/articles/10.3389/fpsyg.2022.945951/full#supplementary-material
