# Supplementary material for: Attribute analysis and modeling of color harmony based on multi-color feature extraction in real-life scenes
Source: Front Psychol. 2022 Sep 14;13:945951. doi: 10.3389/fpsyg.2022.945951 (PMC9518642; doi:10.3389/fpsyg.2022.945951)
Supplement: Supplementary file 1 [file Presentation_1.pdf]

Source Code for Multi-color Physical Features Extraction based on Matlab Simulation

Shuang Wang[1,2,3], Jingyu Liu[1,2,3], Jian Jiang[4], Yujian Jiang[1,2,3*], Jing Lan[5]

[1] *State Key Laboratory of Media Convergence and Communication, Communication University of China, Beijing, China*

[2]*Key Laboratory of Acoustic Visual Technology and Intelligent Control System (Communication University of China), Ministry of Culture and Tourism, Beijing, China*

[3]*Beijing Key Laboratory of Modern Entertainment Technology (Communication University of China) , Beijing, China*

[4]*China Digital Culture Group Co., Ltd, Beijing, China*

[5]*Center for Ethnic and Folk Literature and Art Development, Ministry of Culture and Tourism, Beijing, China*

Corresponding author's e-mail: yjjiang@cuc.edu.cn

Table of contents

feature_extraction_color_moment.m 3

feature_extraction_color_richness.m 4

feature_extraction_space_density.m 5

feature_extraction_color_tone_contrast.m 7

feature_extraction_lightness_ contrast.m 8

feature_extraction_cool/warm_ contrast.m 9

feature_extraction_area_ difference.m 10

feature_extraction_color_moment.m

```
{
    img=imread(name);
    lab=rgb2lab(img);

    L=lab(:,:,1);
    a=lab(:,:,2);
    b=lab(:,:,3);

    [m,n,~]=size(img);

    data_L=reshape(L,m*n,1);
    data_a=reshape(a,m*n,1);
    data_b=reshape(b,m*n,1);


    f_1=mean(data_L);
    f_2=mean(data_a);
    f_3=mean(data_b);

    f_4=std(data_L);
    f_5=std(data_a);
    f_6=std(data_b);

    f_7=mean((data_L-mean(data_L)).^3)^(1/3);
    f_8=mean((data_a-mean(data_a)).^3)^(1/3);
    f_9=mean((data_b-mean(data_b)).^3)^(1/3);

    color_moment=[f_1,f_2,f_3,f_4,f_5,f_6, f_7,f_8,f_9];
}
```

feature_extraction_color_richness.m

```
{
    img=imread(name);
    img=rgb2gray(img);

    [M,N,~]=size(img);

    temp=zeros(1,256);

    for m=1:M
        for n=1:N
            if A(m,n)==0;
                 i=1;
             else
                 i=A(m,n);
             end
             temp(i)=temp(i)+1;
         end
    end

    temp=temp/(M*N);
    result=0;
    for i=1:length(temp)
        if temp(i)==0
        else
            result=result-temp(i)*log2(temp(i));
        end
    end

    f_10=result;

    color_richness=[f_10];
}
```

feature_extraction_space_density.m

{

```
rgb=imread(name);

if ndims(rgb) == 3
    I = rgb2gray(rgb);
else
    I = rgb;
end

hy = fspecial('sobel');
hx = hy';
Iy = imfilter(double(I), hy, 'replicate');
Ix = imfilter(double(I), hx, 'replicate');
gradmag = sqrt(Ix.^2 + Iy.^2);

L = watershed(gradmag);
se = strel('disk', 20);

Io = imopen(I, se);
Ie = imerode(I, se);
Iobr = imreconstruct(Ie, I);
Ioc = imclose(Io, se);
Iobrd = imdilate(Iobr, se);
Iobrcbr = imreconstruct(imcomplement(Iobrd), imcomplement(Iobr));
Iobrcbr = imcomplement(Iobrcbr);

fgm = imregionalmax(Iobrcbr);

It1 = rgb(:, :, 1);
It2 = rgb(:, :, 2);
It3 = rgb(:, :, 3);
It1(fgm) = 255;
It2(fgm) = 0;
It3(fgm) = 0;
I2 = cat(3, It1, It2, It3);

se2 = strel(ones(5,5));
fgm2 = imclose(fgm, se2);
fgm3 = imerode(fgm2, se2);
fgm4 = bwareaopen(fgm3, 20);

It1 = rgb(:, :, 1);
```

```
    It2 = rgb(:, :, 2);
    It3 = rgb(:, :, 3);
    It1(fgm4) = 255; It2(fgm4) = 0; It3(fgm4) = 0;
    I3 = cat(3, It1, It2, It3);

    bw = im2bw(Iobrcbr, graythresh(Iobrcbr));
    D = bwdist(bw);
    DL = watershed(D);
    bgm = DL == 0;

    gradmag2 = imimposemin(gradmag, bgm | fgm4);
    L = watershed(gradmag2);

    [m,n]=size(L);
    L=reshape(L,m*n,1);
    tab=tabulate(L);
    f_11=length(tab(:,1));
    f_12=std(tab(:,2));

    space_density=[f_11,f_12];
}
```

feature_extraction_color_tone_contrast.m

```
{
    img=imread(name);

    [m,n,~]=size(img);

    R=img(:,:,1);
    G=img(:,:,2);
    B=img(:,:,3);

    [L,a,b]=rgb2lab(R,G,B);
    data_a=reshape(a,m*n,1);
    data_b=reshape(b,m*n,1);

    mean_a=mean(data_a);
    mean_b=mean(data_b);
    sum=0;
    for i=1:m
        for j=1:n
            sum=sum+((a(i,j)-mean_a).^2+(b(i,j)-mean_b).^2);
        end
    end
    f_13=((1/(m*n))*sum)^(1/2);

    color_tone_contrast=[f_13];
}
```

feature_extraction_lightness_ contrast.m

```
{
    img=imread(name);
    img=rgb2lab(img);

    L=img (:,:,1);
    a=img (:,:,2);
    b=img (:,:,3);

    [m,n,~]=size(img);

    g = padarray(L,[1 1],'symmetric','both');

    [r,c] = size(g);
    g = double(g);
    k=0;
    for i=2:r-1
        for j=2:c-1
               k = k+(g(i,j-1)-g(i,j))^2+(g(i-1,j)-
        g(i,j))^2+(g(i,j+1)-g(i,j))^2+(g(i+1,j)-g(i,j))^2+(g(i-1,j-1)-
        g(i,j))^2+(g(i-1,j+1)-g(i,j))^2+(g(i+1,j-1)-
        g(i,j))^2+(g(i+1,j+1)-g(i,j))^2;
        end
    end

    f_14 = k/(8*(m-2)*(n-2)+6*(2*(m-2)+2*(n-2))+4*3);

    lightness_contrast=[f_14];
}
```

feature_extraction_cool/warm_ contrast.m

```
{
    img=imread(name);
    img=rgb2lab(img);

    L=img (:,:,1);
    a=img (:,:,2);
    b=img (:,:,3);

    [m,n,~]=size(img);
    data_L=reshape(L,m*n,1);
    data_a=reshape(a,m*n,1);
    data_b=reshape(b,m*n,1);

    for k=1:m*n
        if data_a(k)==0
            data_h(k)=0;
        else
            data_h(k)=atand(data_b(k)/data_a(k));
        end
        data_C(k)=sqrt((data_a(k))*(data_a(k))+(data_b(k))*(data_b(k))
    );
        wc(k)=-0.89+0.52*data_C(k)*(cos(data_h(k)-
        50)+0.16*cos(2*data_h(k)-350));
    end

    [r,c] = size(wc);
    g = double(g);
    k=0;
    for i=2:r-1
        for j=2:c-1
                k = k+(g(i,j-1)-g(i,j))^2+(g(i-1,j)-
        g(i,j))^2+(g(i,j+1)-g(i,j))^2+(g(i+1,j)-g(i,j))^2+(g(i-1,j-1)-
        g(i,j))^2+(g(i-1,j+1)-g(i,j))^2+(g(i+1,j-1)-
        g(i,j))^2+(g(i+1,j+1)-g(i,j))^2;
        end
    end

    f_15 = k/(8*(m-2)*(n-2)+6*(2*(m-2)+2*(n-2))+4*3);

    cool/warm_contrast=[f_15];
}
```

feature_extraction_area_ difference.m

```
{
    img=imread(name);
     [m, n, p] = size(img);
    I=single(img);

    k =8;
    G=reshape(I,[m*n,p]);
    [idx,cen,sumD,D]= kmeans(G,k,'distance','sqeuclidean');

    B=zeros(1,8);
    for i=1:8
        B(1,i)=sum(idx(:)==i);
    end

    f_16=std(B);

    F=[f_16];
}
```
